# Integrated Transcriptome and Multiple Activated Pathways in Endometrial Cancer

**DOI:** 10.3389/fgene.2021.680331

**Published:** 2021-12-03

**Authors:** Qi Jin, Xiaohua Jiang, Xin Du, Weiping Hu, Shun Bai, Xian Wang, Bo Xu, Weidong Zhao

**Affiliations:** ^1^ Cheeloo College of Medicine, Shandong University, Jinan, China; ^2^ Division of Life Sciences and Medicine, Reproductive and Genetic Hospital, The First Affiliated Hospital of USTC, University of Science and Technology of China, Hefei, China; ^3^ Reproductive Medicine Center, 901th Hospital of PLA Joint Logistic Support Force, Hefei, China; ^4^ Division of Life Sciences and Medicine, Department of Obstetrics and Gynecology, The First Affiliated Hospital of USTC, University of Science and Technology of China, Hefei, China; ^5^ Department of Pathology, The Second Affiliated Hospital of Anhui Medical University, Hefei, China

**Keywords:** endometrial cancer, transcriptome sequencing, enrichment analysis, TGF- β pathway, PI3K-Akt pathway

## Abstract

Because the incidence of endometrial cancer is notably increasing worldwide, it has become the leading gynecologic cancer in the United States. Standard treatment results in the loss of reproductive function in women of childbearing age. Furthermore, advanced cancer stages are associated with poor overall survival. The aim of this study was to explore the abnormal expression profile of genes during the development of endometrial cancer, which is essential to provide a better understanding of the mechanisms involved. Five pairs of endometrial cancer tissues and normal endometrial tissues were subjected to next-generation transcriptome sequencing technology. Quantitative real-time PCR (RT-qPCR) was performed to validate the expression profile of key differentially expressed genes (2.0-fold change, adj. *p* < 0.05) (DEGs) identified in the RNA-seq result. GO and KEGG pathways were used for bioinformatic analyses. The transcriptomic sequencing results showed 1153 DEGs, including 673 upregulated and 480 downregulated genes, in the EC specimens. Decreased expression of ID1, IGF1, GDF7, SMAD9, TGF-beta and WNT4, as well as GDF5, INHBA and ERBB4 overexpression, were confirmed in EC using RT-qPCR. Additionally, EC tissue exhibited marked enrichment in genes promoting cellular adhesion, proliferation, migration and plasma membrane. KEGG analysis revealed changes in various pathways, such as the TGF-beta, PI3K-Akt, Wnt, and estrogen pathways. Our data describe the molecular events involved in the pathogenesis of EC, which may be potential diagnostic markers and targets of therapeutic interventions.

## Introduction

According to China National Cancer Center in 2019, the incidence of endometrial cancer in China was 10.28/100,000, and the mortality rate was 1.9/100,000. In the USA, approximately 110,070 new cases of endometrial cancer (EC) were diagnosed in 2018, and it is considered the fourth most common type of cancer after breast, lung and colorectal carcinoma (Lortet-Tieulent et al., 2018; Siegel et al., 2021). Although approximately 90% of EC cases are diagnosed in women aged 50–55 years, approximately 21% of affected women receive an EC diagnosis before menopause (Tohma et al., 2018). EC is the most common gynecologic malignancy, and various risk factors, such as excessive exposure of the endometrium to estrogen, early menarche, late menopause, tamoxifen therapy, nulliparity, infertility and polycystic ovary syndrome, have been associated with the onset of EC. Additional risk factors for EC include obesity, hypertension, diabetes and Lynch syndrome (Siegel et al., 2018).

With increases in the childbearing age, traditional surgical methods provide no opportunity for fertility preservation. Approximately 75% of women presenting with early-stage endometrial cancer could be taken under consideration with strict indications and follow-up for fertility-sparing treatment (McKenzie et al., 2018). However, advanced stages of EC may result in a poor prognosis; thus, early detection and treatment is the primary strategy for managing endometrial cancer.

In this decade, next-generation sequencing (NGS) technologies have enabled clinicians and researchers to readily evaluate the transcriptome profile of various species to investigate dynamic biological processes with a high level of sensitivity and accuracy (Martin and Wang, 2011). Subsequently, bioinformatics analysis through Gene Ontology (GO) and Kyoto Encyclopedia of Genes and Genomes (KEGG) pathways can reveal the underlying biological processes, cellular components and molecular functions for elucidating the potential etiology and prognostic factors of diverse types of cancers. A previous study demonstrated the application of an integrated genomic, transcriptomic and proteomic characterization of EC and classified it into four categories that may impact adjuvant treatment after surgery for late-stage women (Cancer Genome Atlas Research et al., 2013).

In the current study, we utilized RNA sequencing and bioinformatics analysis of five pairs of endometrial cancer and normal endometrial tissue to comprehensively investigate the differentially expressed genes. Next, we selected a well-known pathway that has an association with EC etiology and further validated it by RT-qPCR. Thus, our findings may provide a new perspective for future research aimed at diagnosing EC in an early stage and identifying some novel targets for treating EC.

## Materials and Methods

### Sample Collection

A total of 53 EC patients who were treatment-naive prior to surgery and 27 normal cases were recruited in this study. Total RNA from five pairs of cancerous and normal endometrial tissues was randomly selected for transcriptome sequencing. The samples were collected in June 2019 and January 2020 at the Department of Obstetrics and Gynecology of the First Affiliated Hospital of University of Science and Technology of China. This study was performed after approval from the Ethics Committee on Human Research of the First Affiliated Hospital of the University of Science and Technology of China. All sampling and experimental procedures were performed by strictly following the ethical standards of the 1964 Declaration of Helsinki and its latest amendments or comparable ethical standards. Informed written consent was obtained from all enrolled individuals. Each specimen was separated into two parts: one part was immediately placed in 10% neutral formalin fixative, and the other was quickly stored at −80°C.

### Histological Analysis

Immunohistochemical staining was performed on formalin-fixed, paraffin-embedded tissue sections. All cases were cut into 4-μm histological sections and stained with H&E and commercially available antibodies. The slides were incubated with antibodies against CK7 (working concentration, Zhong Shan Goldenbridge Biotechnology, Beijing, China), Vim (working concentration, Zhong Shan Goldenbridge Biotechnology, Beijing, China), PCNA (working concentration, Zhong Shan Goldenbridge Biotechnology, Beijing, China) and β-catenin (dilution 1:100, Bioss, Beijing, China). Heat-induced antigen retrieval was carried out in a pressure cooker in citrate buffer solution (pH 6.0). The sections were counterstained with hematoxylin, dehydrated, and mounted. The immunohistochemical reactions were visualized under an Olympus microscope (OLYMPUS CX43, Tokyo, Japan). Appropriate positive and negative controls for each antibody were also stained. By multiplying the staining intensity (SI) and the percentage of positive cells (PP), a score ranging from 0 to 12 was established. SI was classified as no reaction (0 points), slightly stained (1 point), moderately stained (2 points) and strongly stained (3 points). PP was evaluated at five levels: >80% of positive (4 points), 51–80% positive cells (3 points), 10–50% positive cells (2 points), <10% (one point) and negative (0 point). A score >3 was defined as a positive reaction, and 9 points was defined as strongly positive.

### RNA Extraction and Quality Assessment

Total RNA extraction was performed with TRIzol reagent (Life Technologies, CA, United States) according to the manufacturer’s instructions. The quality and quantity of the extracted RNA was assessed using a NanoDrop ND-1000 (Wilmington, DE, United States). A Bioanalyzer 2100 RNA-6000 Nano Kit and Agilent 2100 Bioanalyzer (Agilent Technologies, Santa Clara, CA, United States) were used to evaluate the RNA integrity. Samples exhibiting an RNA integrity number (RIN) > 7 were used for subsequent analysis.

### Library Preparation for Transcriptome Sequencing

Three micrograms of total RNA per sample was utilized for making the sequencing libraries, which were generated using the NEBNext Ultra RNA Library Prep Kit of Illumina (NEB, United States) following the manufacturer’s instructions. The RNA fragments were reverse transcribed into first-strand cDNA using random hexamer primers, while DNA Polymerase I was employed to synthesize double-stranded cDNA. The AMPure XP system (Beckman Coulter) was used to purify the cDNA fragments. The libraries were constructed using TruSeq Stranded Total RNA with Ribo-Zero Gold according to the manufacturer’s instructions. Then, these libraries were sequenced on the Illumina sequencing platform (HiSeq^TM^ 2500 or other platform), and 150 bp/125 bp paired-end reads were generated.

### Bioinformatics Analysis: GO and KEGG Pathway Analysis

The estimated SizeFactors function of the DESeq (2012) R package was employed to capture the DEGs. Gene Ontology (GO) analysis involves three levels of functional gene attributes, associating differentially expressed mRNAs with GO categories derived from Gene Ontology (http://www.geneontology.org). Kyoto Encyclopedia of Genes and Genomes (KEGG) pathway analysis for differentially expressed mRNAs (DE mRNAs) was performed using the latest KEGG (https://www.genome.jp/kegg) database, which provided the biological pathways of the significantly enriched mRNAs. Using nbinomTest, DEGs with *p* < 0.05 and fold-change values larger than two were selected. The differential mRNA GO and KEGG enrichment were analyzed by a hypergeometric distribution test.

### Quantitative Real-Time PCR

Ten genes were selected to perform RT-qPCR with another seven pairs of endometrial cancer tissue and normal endometrial tissue for validation of the transcriptome sequencing. The reaction system consisted of 5 μL SYBR PREmis EX Taq^TM^ (2x), 0.4 μL forward primer (10 μM), 0.4 μL reverse primer (10 μM), 1 μL cDNA, 0.2 μL 50 × ROX Reference Dye, and 3 μL RNase-Free ddH2O (Xu et al., 2011). First-strand cDNA was synthesized using a Biosystems StepOne Real-Time PCR System (Applied Biosystems, Foster City, CA, United States) and amplified by real-time qPCR with a SYBR premix Ex Taq II kit (Takara). The PCRs were performed in triplicate wells at 95°C for 30 s, followed by 40 cycles of denaturing at 95°C for 5 s and 60°C for 31 s. All data were normalized to β-actin. DEG expression levels were calculated using the 2−ΔΔCT method. All RT-qPCR primers were designed using Primer6.0 based on the gene sequences reported in the NCBI database.

### Statistical Analysis

In this study, the RT-qPCR was repeated at least three times, and the quantitative data were compared using Student’s t-tests. *p* < 0.05 was considered significant (SPSS 20, mean + SD). For real-time PCR analysis, Cт values of samples were normalized to the corresponding Cт values of β-actin. Quantification of the fold change in gene expression was determined by the comparative Cт method.

## Results

### Clinicopathological Characteristics and Immunohistochemistry

**FIGURE 1:**
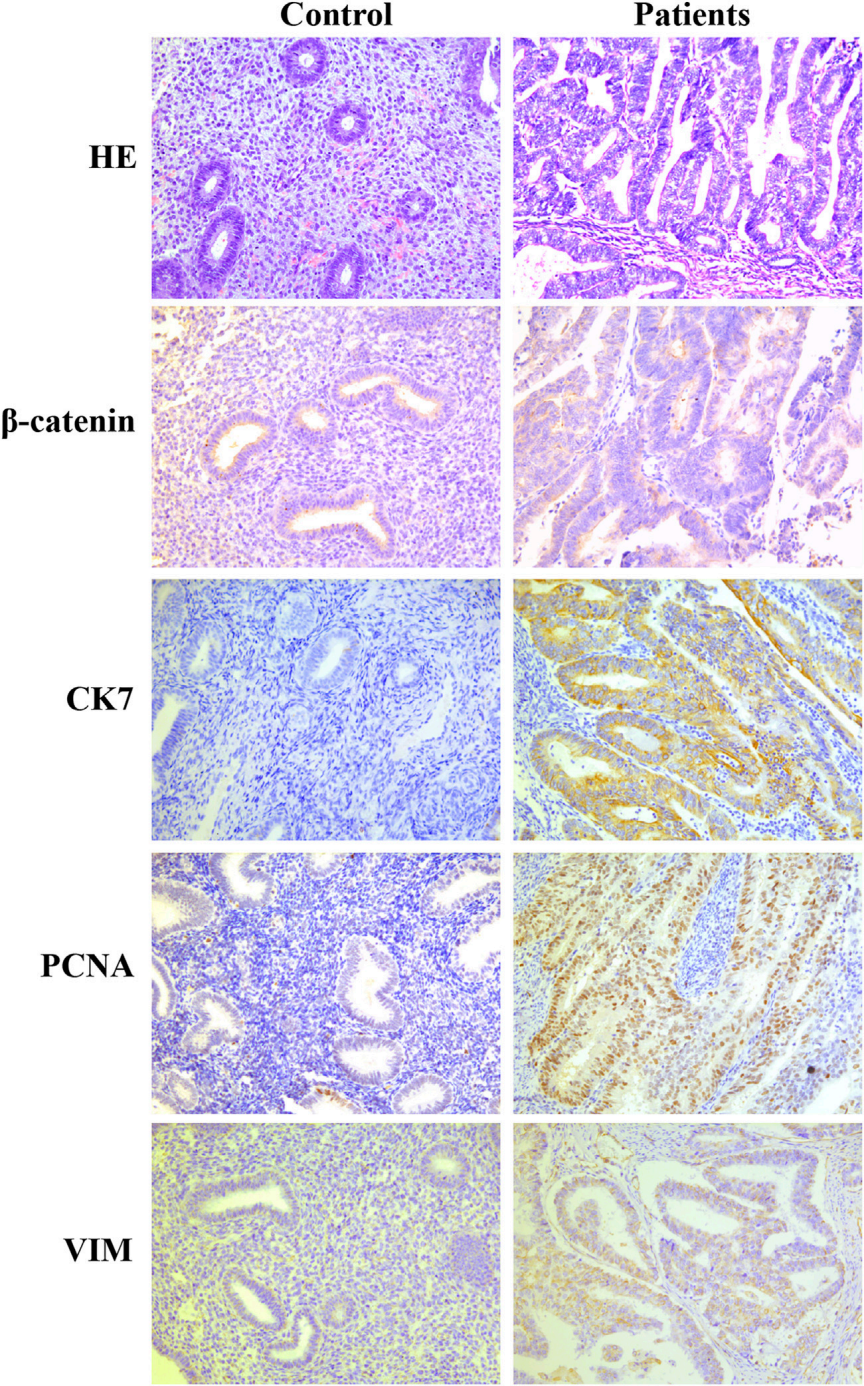
Immunohistochemical markers of PCNA, CK7, Vim and β-catenin were expressed at higher levels in endometrial cancer tissues than in the control group.

The clinical information and tumor characteristics of the five patients are summarized in Table 1. PCNA staining revealed their location in the nucleus. Subsequently, immunohistochemical staining for CK7 and Vim detected them in the cytoplasm and membranes of glandular cells. The expression of β-catenin was observed in the cytoplasm of glandular cells. Both immunohistochemical markers were expressed at higher levels in endometrial cancer tissues than in the control group (Figure 1). PCNA expression was found to be related to pathogenesis and prognosis of EC patients.

**TABLE 1:** Clinical-pathologic characteristics of patients.

Patient ID	Age	Pathological type	Histological grade	TNM stage	Tumour size	Depth of myometrial invasion	Lymph node metastasis
EC 4	55	Endometrioid carcinoma	Moderately differentiated	IIIC2	>2 cm	<1/2	Yes
EC 6	53	Endometrioid carcinoma	Moderately differentiated	II	>2 cm	<1/2	No
EC 9	61	Endometrioid carcinoma	Poorly differentiated	II	<2 cm	<1/2	No
EC 19	53	Endometrioid carcinoma	Well differentiated	IA	>2 cm	<1/2	No
EC 20	41	Endometrioid carcinoma	Well differentiated	IB	>2 cm	≥1/2	No

### Transcriptome Data

Based on the filtered sequencing reads, we obtained 26.36 G clean data from the transcriptome sequencing. The effective data volume of each sample was distributed at 13.09–13.27 G, Q30 bases were in the range of 92.38–93.41%, and the average GC content was 46.73%. The reads were compared with the reference genome, and the genome comparison of each sample was obtained with a comparison rate of 97.73–97.97%. Overall, 1153 different genes were annotated from the transcriptome data.

### Identification and Analysis of the DEGs

In total, 1153 DEGs (673 upregulated genes and 480 downregulated genes) that were differentially expressed in endometrial cancer tissue compared with normal endometrial tissue were detected (|Log2FC| >1) (Supplementary Table S1). The top 20 upregulated genes are listed in Table 2, while the top 20 downregulated genes are shown in Table 3.

**TABLE 2:** Top 20 upregulated genes.

Gene_id	log2FoldChange	pValue	Product
DKK4	9.49490016254192	1.04E-21	Dickkopf WNT signaling pathway inhibitor 4
CXCL5	7.06885630360606	1.98E-18	C-X-C motif chemokine ligand 5
KRT6A	8.40409722553361	5.51E-18	Keratin 6A
JCHAIN	6.06198197453528	6.50E-17	Joining chain of multimeric IgA and IgM
IGLL5	5.8619180160352	4.14E-14	Immunoglobulin lambda like polypeptide 5
MYH11	5.06142411741398	5.25E-13	Myosin heavy chain 11
DES	5.77609393749455	2.15E-12	Desmin
GRIA2	4.73036064253495	4.74E-12	Glutamate ionotropic receptor AMPA type subunit 2
CAPN13	5.35977088766126	1.37E-10	Calpain 13
TMC5	4.1761744996929	3.41E-10	Transmembrane channel like 5
LTF	4.09383816510685	6.29E-10	Lactotransferrin
CXCL9	4.88148392061089	8.35E-10	C-X-C motif chemokine ligand 9
CFB	3.87428570966326	1.73E-09	Complement factor B
HLA-DRB5	3.94547877462524	1.93E-09	Major histocompatibility complex, class II, DR beta 5
CHL1	4.12848580242201	2.38E-09	Cell adhesion molecule L1 like
TFAP2A	5.88842619477027	2.53E-09	Transcription factor AP-2 alpha
SPP1	3.82435858149562	3.59E-09	Secreted phosphoprotein 1
COL8A1	5.77407427103097	4.62E-09	Collagen type VIII alpha 1 chain
S100A9	4.45915743773891	9.05E-09	S100 calcium binding protein A9
HS3ST3A1	6.08509931162922	9.93E-09	Heparan sulfate-glucosamine 3-sulfotransferase 3A1

**TABLE 3:** Top 20 downregulated genes.

Gene_id	log2FoldChange	pValue	Product
EPHA5	-7.17216432676385	9.64E-16	EPH receptor A5
MMP26	-6.12022585097862	1.59E-14	Matrix metallopeptidase 26
PENK	-8.34808303279188	8.70E-14	Proenkephalin
NRXN1	-7.43538544089878	4.04E-13	Neurexin 1
KIAA1210	-4.85573980734559	1.25E-11	KIAA1210
CACNA1G	-4.67635324042015	2.63E-11	Calcium voltage-gated channel subunit alpha1 G
P2RY14	-4.35141244693077	5.57E-10	Purinergic receptor P2Y14
OGN	-3.97502184228362	8.89E-10	Osteoglycin
ROBO3	-5.28296919609017	9.48E-10	Roundabout guidance receptor 3
ADAMTS16	-4.19653440305854	1.12E-09	ADAM metallopeptidase with thrombospondin type 1 motif 16
PKD1L2	-4.37397769545082	1.17E-09	Polycystin 1 like 2 (gene/pseudogene)
CCBE1	-4.02495760723948	4.39E-09	Collagen and calcium binding EGF domains 1
NLGN1	-4.16419236236428	5.62E-09	Neuroligin 1
VWC2	-4.89442595363418	7.97E-09	Von Willebrand factor C domain containing 2
PCSK5	-3.65263455895927	1.20E-08	Proprotein convertase subtilisin/kexin type 5
ECM1	-3.78147598317963	1.83E-08	Extracellular matrix protein 1
PGBD5	-4.58749330903002	1.91E-08	PiggyBac transposable element derived 5
MMP16	-3.50208469660249	3.69E-08	Matrix metallopeptidase 16
OVGP1	-3.47702587273636	4.79E-08	Oviductal glycoprotein 1
OLFM1	-3.85033591812851	6.95E-08	Olfactomedin 1

### GO and KEGG Pathway Enrichment Analysis

**FIGURE 2:**
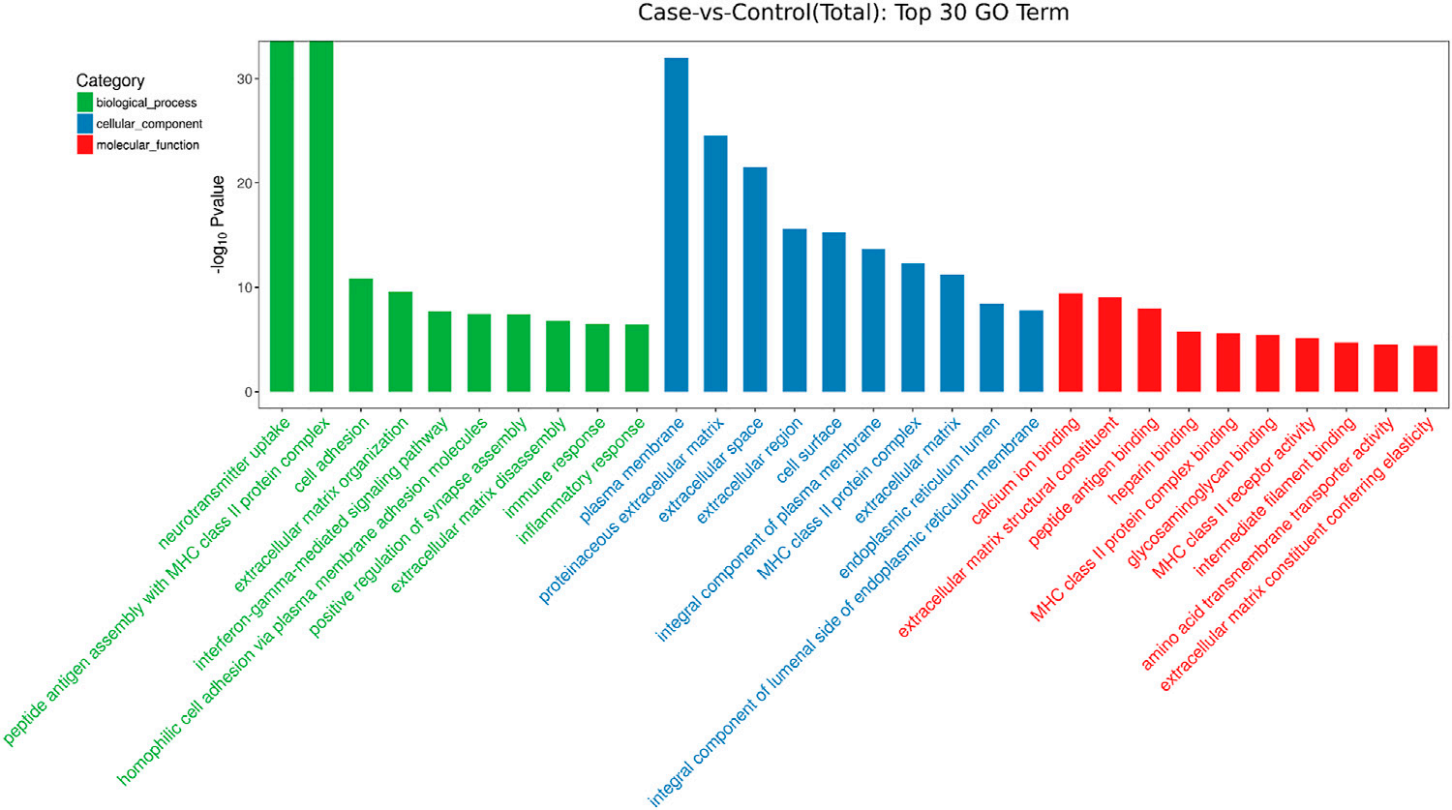
The top 30 GO terms of total between endometrial cancer and normal endometrium.

**FIGURE 3:**
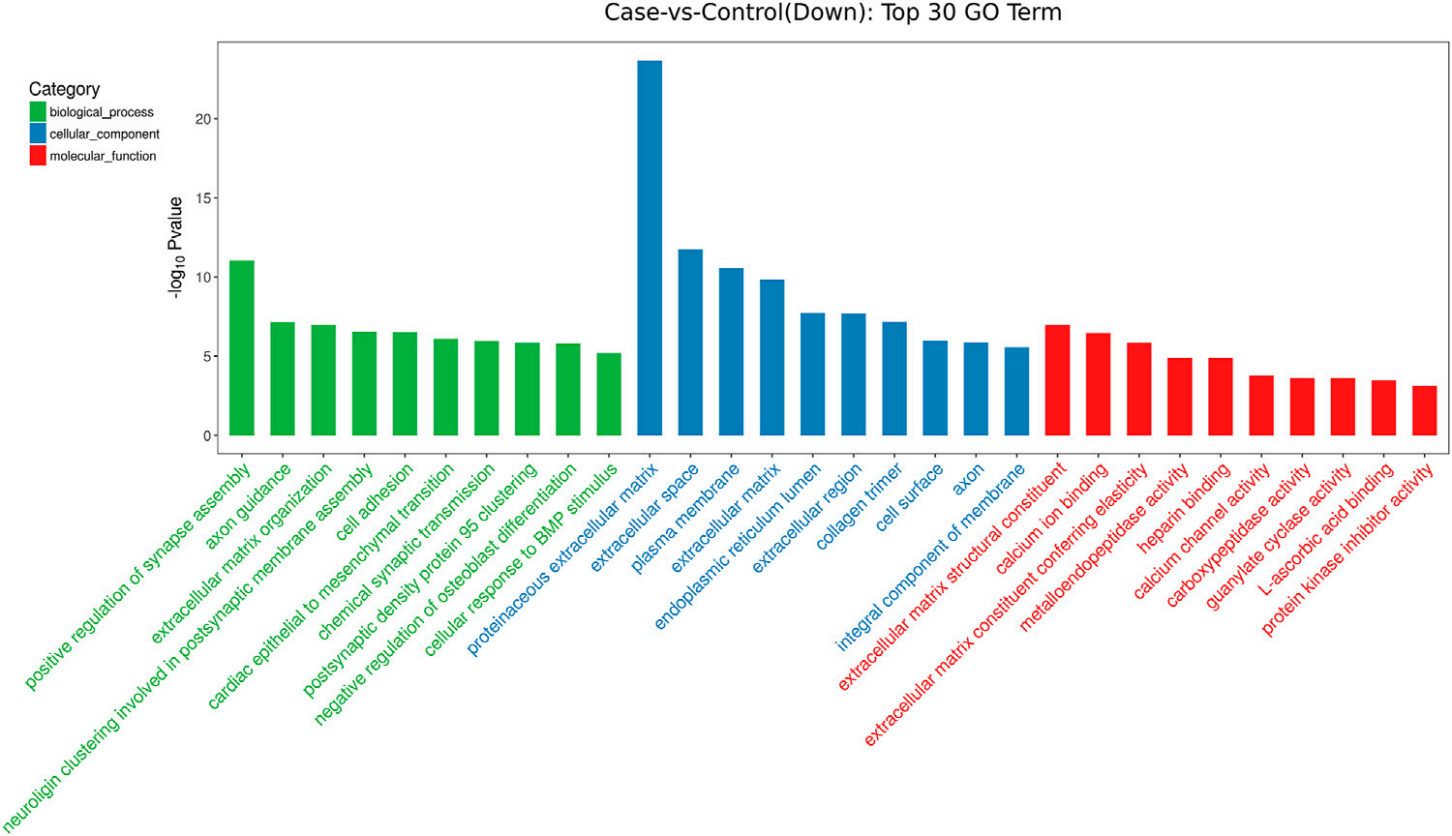
The top 30 GO terms of down between endometrial cancer and normal endometrium.

**FIGURE 4:**
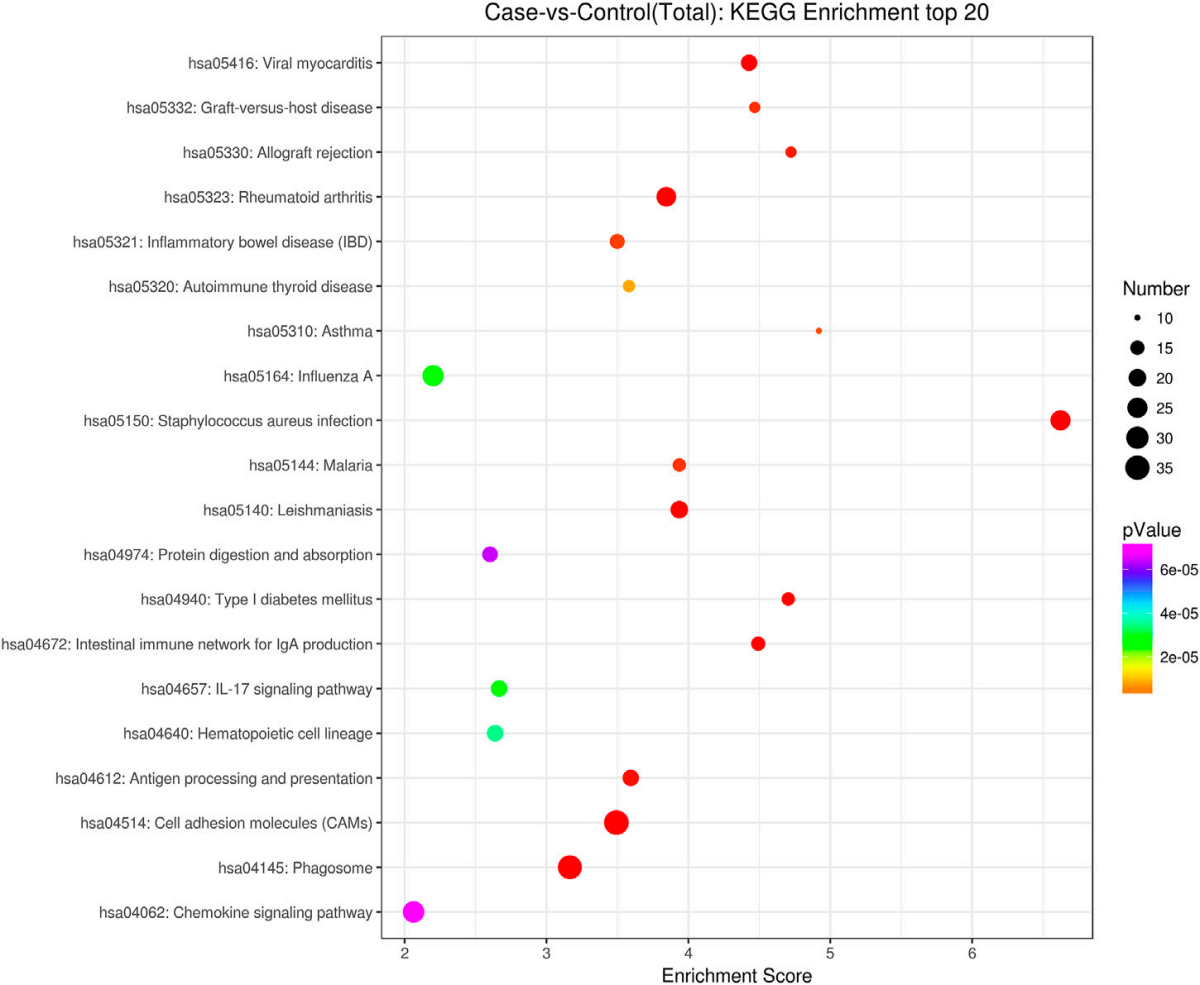
KEGG pathway analysis of the total top 20 pathways between endometrial cancer and normal endometrium.

**FIGURE 5:**
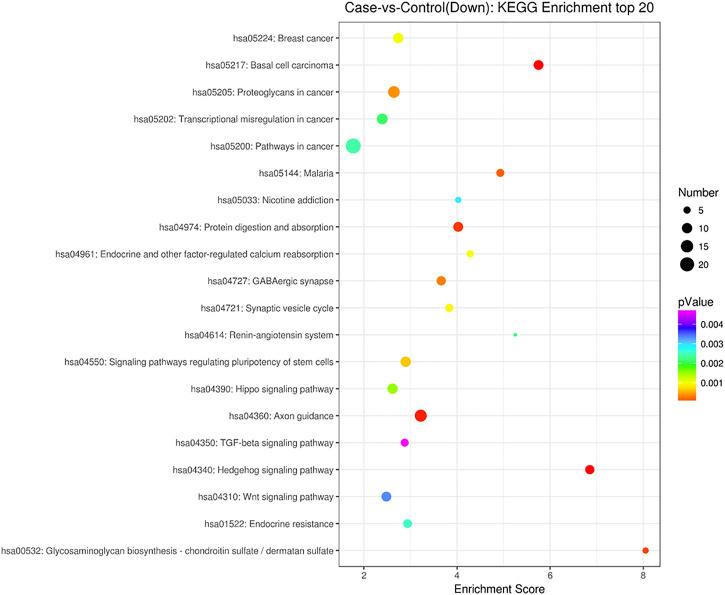
KEGG pathway analysis of the down top 20 pathways between endometrial cancer and normal endometrium.

GO analysis showed that these DEGs are mainly involved in neurotransmitter uptake (biological process), plasma membrane (cellular component), and calcium ion binding (molecular function). The top 30 GO terms of total and down are listed in Figures 2, 3. KEGG pathway analysis revealed that the top 20 pathways were enriched, such as cell adhesion molecules (CAMs), phagosomes, and *Staphylococcus aureus* infection (Figure 4). Among them, protein digestion and absorption, axon gauidance, and basal cell carcinoma were the most enriched among the top 20 downregulated genes (Figure 5).

### RT-q PCR to Validate the Transcriptome Expression

**FIGURE 6:**
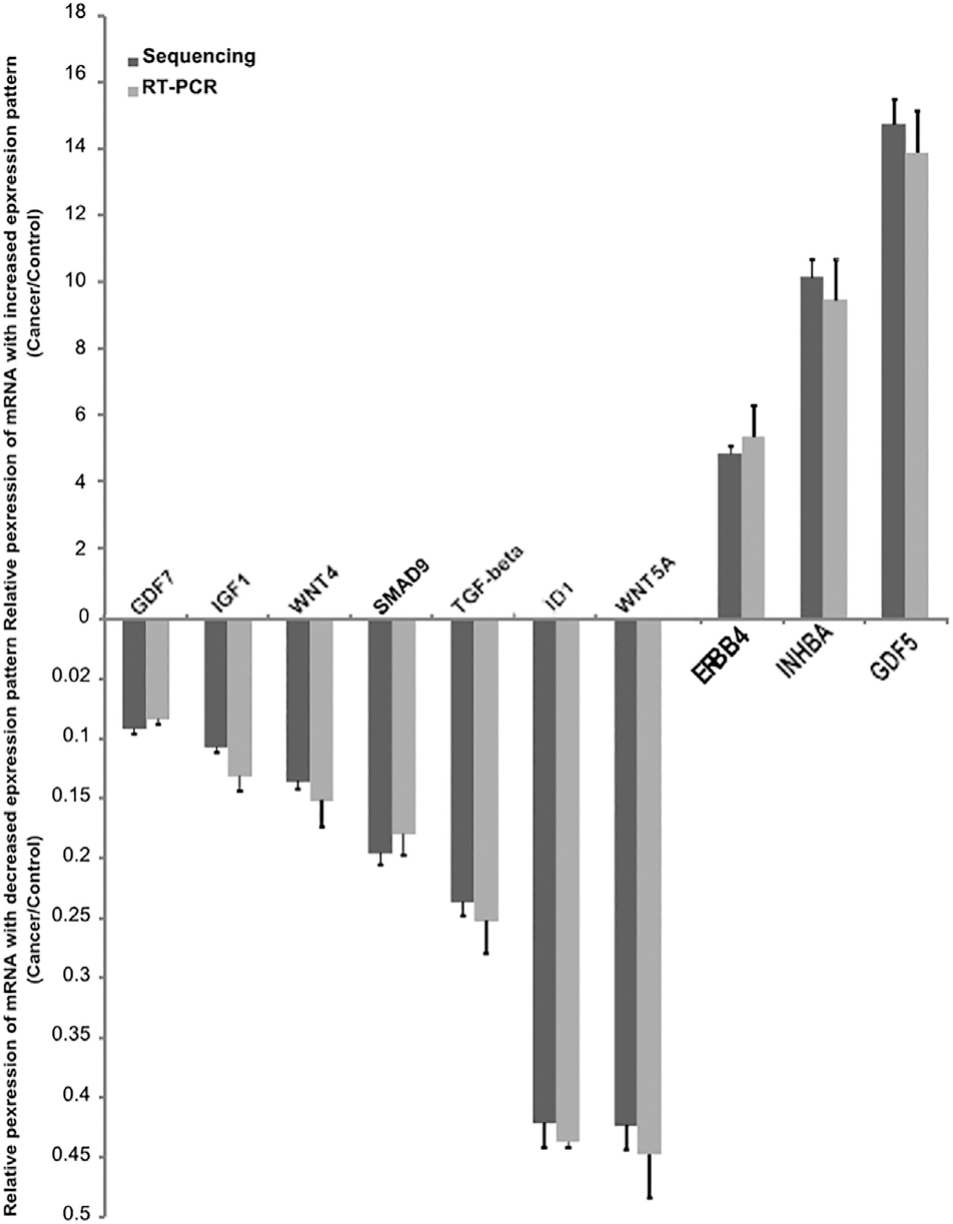
Confirmation of the differentially expressed mRNAs between cancer and control endometrium tissue. Validation of the expression of 10 mRNAs by quantitative real-time PCR, showing increased expression of mRNAs (3) and decreased mRNA (7). The real-time PCR data with bars represent a mean ± SD from 3 independent experiments, and the sequencing data with bars represent the 95% CI.

We selected 10 important genes, namely inhibitor of differentiation-1 (ID1), growth differentiation factor 7 (GDF7), growth differentiation factor 5 (GDF5), inhibin-β A (INHBA), sterile alpha motif domain-containing protein 9 (SMAD9), transforming growth factor-beta (TGF-beta), Wnt family member 4 (WNT4), Wnt family member 5A (WNT5A), erb-B2 receptor tyrosine kinase 4 (ERBB4), and insulin-like growth factor 1 (IGF1), from the RNA-seq data and subsequently validated them using RT-q PCR. The RT-qPCR results demonstrated concordant expression with the transcriptome sequencing results (Figure 6). All of the primers used in this study are listed in Table 4.

**TABLE 4:** qRT-PCR primer sequences.

Gene symbol	Forward primer (5–3′)	Reverse primer (5–3′)
ID1	CGT​GCT​GCT​CTA​CGA​CAT​GA	CGA​CAC​AAG​ATG​CGA​TCG​TCC
GDF7	TGA​TGT​CGC​TTT​ACC​GGA​GC	TGG​ACA​CGT​CGA​ACA​GGA​AG
GDF5	CAC​GAG​AAA​GCC​CTG​TTC​CT	CCA​GCC​CAT​GTC​CTT​GAA​GT
INHBA	GGA​GTG​TGA​TGG​CAA​GGT​CA	ACA​TGG​GTC​TCA​GCT​TGG​TG
TGF-beta	ATG​GTT​GTT​TCC​AGT​TTG​GTC​AC	CAA​GGA​ACT​TCA​CAA​GAG​CAG​TC
WNT4	TCG​TCT​TCG​CCG​TCT​TCT​CAG	GAG​TCG​AGT​GTG​GAG​CAG​TT
SMAD9	AGA​CAT​TCC​AGG​CTT​CCT​CC	ATA​GTT​GCA​GTT​CCG​GCT​CT
WNT5A	TTT​GGC​AGG​GTG​ATG​CAG​AT	TTG​GCA​AAG​CGG​TAG​CCA​TA
ERBB4	ACA​GGC​TAC​GTG​TTA​GTG​GC	GCA​ACG​TCC​ACA​TCC​TGA​AC
IGF1	ATC​TCT​TCT​ACC​TGG​CGC​TG	CGC​AAT​ACA​TCT​CCA​GCC​TC
Actinb	AAT​GAG​CTG​CGT​GTG​GCT​C	ATA​GCA​CAG​CCT​GGA​TAG​CAA​C

## Discussion

Gynecological malignancies can affect women of reproductive age, whose fertility preservation is a priority. Nearly 14% of endometrial cancer cases affect women of childbearing age (McKenzie et al., 2018). Additionally, a significant proportion of women are diagnosed with endometrial cancer prior to pregnancy. Traditionally, the standard of care for these patients includes total hysterectomy (TH) and bilateral salpingo-oophorectomy (BSO) with selective use of pelvic and/or para-aortic lymph node dissection depending on the risk factors and cancer staging (Koh et al., 2018). Recently, a meta-analysis of 1,038 women with a diagnosis of early stage endometrial cancer or complex atypical hyperplasia demonstrated that conservative management with progestins combined with an IUD eventually had a pregnancy rate of 40% (95% CI: 20–63%) and only a 35% rate of live newborns (Wei et al., 2017). Most patients with stage I and II disease have a favorable prognosis, whereas patients with advanced disease (stage III or IV) have a worse likelihood of survival (Lewin et al., 2010). Therefore, our current study provides RNA-Seq transcriptome analysis of tissues in endometrial cancer compared with normal endometrium. Herein, we integrated the power of next-generation sequencing with the pathogenesis of a pathway analysis platform to initially analyze the biology of endometrial cancer and to highlight various functional pathway signaling pathways during endometrial cancer progression.

Our analyses revealed the enrichment of gene signatures of cell proliferation, differentiation, migration and morphogenesis. TGF-β, the transforming growth factor-β superfamily, comprises over 30 members in humans, including TGF-βs, activins, inhibins, nodals, bone morphogenetic proteins (BMPs), growth and differentiation factors (GDFs) and related proteins (David and Massague, 2018; Zhang, 2018). SMAD proteins interact with cell membrane receptors to activate the TGF-β signaling pathway in mammals. SMAD9 is a transcriptional regulator that inhibits BMP signaling in cell culture (Tsukamoto et al., 2014). Downstream BMP-induced signals are mediated by inhibiting Smad1/5/9 expression in human oral squamous cell carcinoma (Chiba et al., 2017). A previous study showed that SMAD9 was significantly downregulated in the normal thyroid compared to the follicular variant of papillary thyroid carcinoma and follicular adenoma (Schulten et al., 2015).

Our data support previous findings suggesting that SMAD9 may inhibit transcriptional activity in endometrial cancer. TGF-β/SMAD signaling is involved in endometrial biological and reproductive functions, which has been proven by mouse model experiments (Kriseman et al., 2019). Other reports demonstrated that decreased TGF-β/SMAD mRNA levels were correlated with the pathogenesis of endometrial cancer (Mhawech-Fauceglia et al., 2011). GDF7 is a ligand in the BMP pathway that has been known to have inhibitory effects on growth in various human cancers. An earlier study found that GDF7 was downregulated in colorectal cancer tissues. In addition, it has been associated with proinflammatory phenotypes and the inflammatory response in both Barrett’s esophagus (Palles et al., 2015) and esophageal adenocarcinoma (Becker et al., 2016). In the current study, we found that GDF7 was also significantly downregulated in endometrial cancer.

The PI3K/AKT pathway plays an essential role in endometrial pathogenesis, which is highly activated in endometrial cancer, often due to PTEN loss (Zaczek et al., 2020). Researchers have suggested that inhibiting the PI3K/AKT pathway and angiogenesis might be an effective therapeutic strategy for the treatment of EC (Liu et al., 2020). Similarly, ERBB4 is one of the members of the epidermal growth factor receptor family, which is firmly linked to cell proliferation and tumor development. ERBB4 could activate related genes in the nucleus, promoting cell division and proliferation. Overexpression of ERBB4 was found to be closely related to poor outcomes and advanced tumor states (Muraoka-Cook et al., 2008) and inhibiting the expression of ERBB4 via the PI3K/Akt signaling pathway reduced the proliferation of gastric cancer cells (Xu et al., 2018).

**FIGURE 7:**
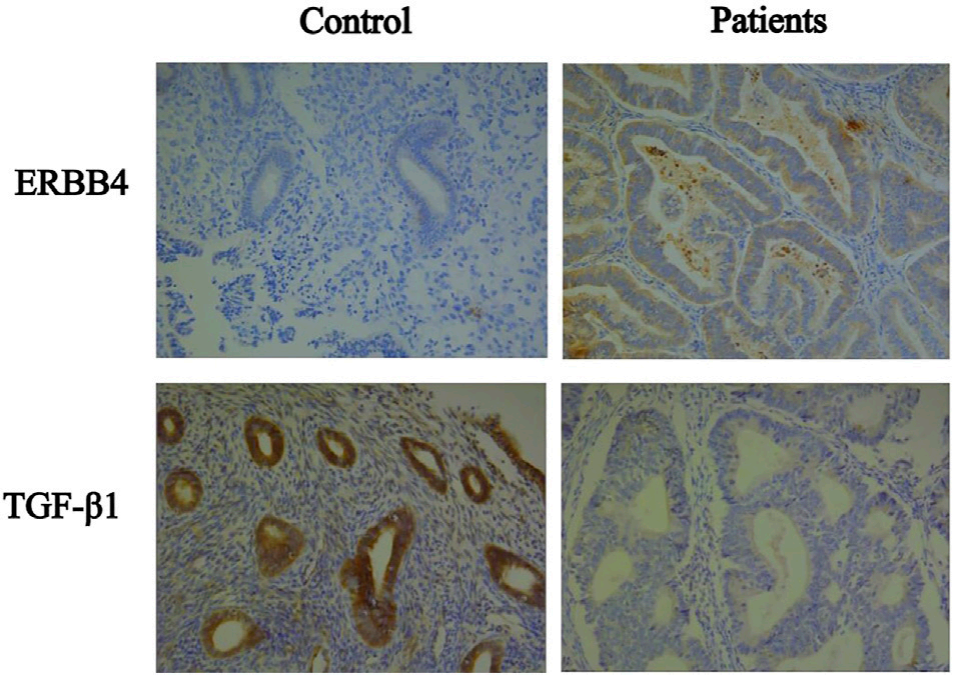
Immunohistochemical of the target protein such as ERBB4 and TGF-β1 between patients and control.

In the present study, we observed that ERBB4 was highly expressed in endometrial cancer compared to normal endometrial tissue; thus, it may provide a clue for endometrial carcinogenesis and may serve as a novel target for treatment. IGF-1 (insulin-like growth factor 1) belongs to the gene family responsible for the regulation of cell proliferation, differentiation, apoptosis and metastasis of cancers (Vetvicka et al., 2016). Overexpression of IGF1 by tumor-associated macrophages promotes the carcinogenesis of epithelial ovarian cancer cells (Liu et al., 2018), whereas our study found a significant downregulation of IGF1 expression in endometrial cancer compared to the control. Similarly, microarray research was consistent with our findings (Hermyt et al., 2019). The controversies around the oncogenic role of IGF-1 can be partly explained by the numerous intracellular signaling pathways affected and the various downstream responses in different cell types. We also validated the expressions of the target protein such as ERBB4 and TGF-β1 in the samples (Figure 7).

The KEGG pathway annotation showed a functional role of the identified molecular networks in EC biology, including the estrogen pathway and Wingless-type (Wnt) pathway, suggesting that multiple signaling pathways are involved in endometrial cancer. Estrogens play a mitogenic role in the normal endometrium, maintaining tissue growth and endometrial receptivity. High levels of estrogens unopposed by progesterone lead to endometrial cancer in animal models, suggesting that the lack of a balance between pro-growth estrogens and anti-growth progestogens may contribute to the early stages of endometrial cancer (Yang et al., 2015). The Wingless-type (Wnt) signaling pathways play critical roles in embryonic development and maintenance of tissue homeostasis. Abnormal Wnt/β-catenin signaling in the endometrium can result in both embryo implantation failure and pathogenic disorders of the endometrium, such as endometriosis and even endometrial cancer (Chen et al., 2019). Deregulation of the Wnt/β-catenin signaling pathway in approximately 10–45% of ECs occurs by inactivating β-catenin mutations and via downregulation of Wnt antagonists by epigenetic silencing (Dellinger et al., 2012). In a recent case-control study in Germany, estrogen signaling and Wnt/β-catenin and systems were dysregulated in EC, showing potential crosstalk between these two pathways, showing the specificity of these molecules in disease characteristics (Kasoha et al., 2020).

In the current work, NGS was used to describe the transcriptome and multiple pathways involved in the pathogenesis of endometrial cancer. The confirmation of the expression profile of genes involved in oncogenic events using RT-qPCR is a strength of this study. In future research, it would be necessary to establish miRNA-mRNA interaction analyses at the protein and cellular levels. Furthermore, developing a comprehensive landscape of the miRNA-mRNA regulatory networks would be possible and could provide useful information for early diagnosis, novel treatments and targeted therapy.

## Data Availability

The datasets presented in this study can be found in online repositories. The names of the repository/repositories and accession number(s) can be found below: NCBI GEO, accession no: GSE182132.
